# Numerical simulation on the effects of drug eluting stents at different Reynolds numbers on hemodynamic and drug concentration distribution

**DOI:** 10.1186/1475-925X-14-S1-S16

**Published:** 2015-01-09

**Authors:** Yu Chen, Yan Xiong, Wentao Jiang, Fei Yan, Meng Guo, Qingyuan Wang, Yubo Fan

**Affiliations:** 1 Department of Applied Mechanics, Sichuan University, Chengdu, 610065, China; 2 School of Manufacturing Science & Engineering, Sichuan University, Chengdu, 610065; 3 School of Biological Science and Medical Engineering, Beihang University, Beijing, 100191, China

**Keywords:** DES, curved artery, secondary flow, Reynolds number, WSS, drug concentration

## Abstract

**Background:**

The changes of hemodynamics and drug concentration distribution caused by the implantation of drug eluting stents (DESs) in curved vessels have significant effects on In-Stent Restenosis.

**Methods:**

A 3D virtual stent with 90°curvature was modelled and the distribution of wall shear stress (WSS) and drug concentration in this model were numerically studied at Reynolds numbers of 200, 400, 600, 800.

**Results:**

The results showed that (1) the intensity of secondary flow at the 45° cross-section was stronger than that at the 90° cross-section; (2) As the Reynolds number increases, the WSS decreases. When the Reynolds number reaches 600, the low-WSS region only accounts for 3% of the total area. (3) The effects of Reynolds number on drug concentration in the vascular wall decreases in proportionally and then the blood velocity increased 4 times, the drug concentration in the vascular wall decreased by about 30%. (4) The size of the high drug concentration region is inversely proportional to the Reynolds number. As the blood velocity increases, the drug concentration in the DES decreases, especially at the outer bend.

**Conclusions:**

It is beneficial for the patient to decrease vigorous activities and keep calm at the beginning of the stent implantation, because a substantial amount of the drug is released in the first two months of stent implantation, thus a calm status is conducive to drug release and absorption; Subsequently, appropriate exercise which increases the blood velocity is helpful in decreasing regions of low-WSS.

## Introduction

Atherosclerosis is a specific form of arteriosclerosis in which an artery wall thickens as a result of the accumulation of cholesterol and triglyceride [1]. Drug eluting stents (DESs) in cardiovascular surgery significantly reduce the rate of restenosis caused by in-stent intimal hyperplasia [2] and have been considered and accepted as one of the most promising treatment methods for preventing restenosis [3].

The blood flow in humans and animals is always laminar, and only in abnormal conditions is there turbulent blood flow for a long time [4]. Qu et al.[5] found that with age the fluctuation ranges of normal value increased and the mean blood velocity decreased. Yin et al.[6] found that the blood velocity in the basilar and carotid arteries in normal humans decreased with age, with the blood velocity in the range 0.2 m-0.8 m/s. Normally, the peak of diastolic velocities in a human coronary artery is 500 ± 170 mm/s, with a mean blood velocity of 370 ± 120 mm/s. With cardio intervention, the average peak velocity (APV) at the proximal and distal increases and the coronary flow reserve (CFR) improves [7]. In addition, the blood velocity for humans when in motion increases up to 30% even higher [8]. From the viewpoint of biomechanics, factors such as blood flow velocity, impact flow, pressure and wall shear stress (WSS) have important effects on atherosclerosis. Seo et al.[9] discussed that how to reduce erratic flow at different Reynolds numbers for different curvature stents. Sukavaneshvar et al.[10] analyzed the hemodynamics on the two restenosis positions at two different Reynolds numbers. Kolachalama et al.[11] discussed the changes of drug concentration, the Area Under Curve(AUC) and the recirculation length at 2, 4, 6 and 8 times Reynolds number. Chen et al.[12] analyzed the effects of drug-coating positions on the drug concentration in curved stents and found that the contribution of the contacting surface to the drug concentration in the vascular wall was underestimated in 2D numerical simulation. The implantation of stents into coronary arteries can influence the fluid dynamics in the regions adjacent to the arterial wall, and consequently cause damage to the regional arterial wall and a change of WSS. Especially when a drug eluting stent (DES) is implanted in a curved vessel, secondary flow arises due to the curvature. Secondary flows occur in the cross-stream direction caused by the base primary flow in the streamwise direction [13]. Secondary flow structures may affect the wall shear stress in arteries, and is closely related to atherogenesis[14]. So the relationships between stent design and placement, drug release, deposition concentration, distribution of low-WSS, and secondary flow are important issues in research on stents. Autumn et al.[15] compared the change of secondary flow, with and without stents, through PIV experiments. Jung et al.[16] thought that the accumulation of red blood cells on the inner curvature was caused by the secondary flow and higher residence times. Bioron et al.[17] analysed the behaviour of unsteady flows in a bend with experimental and numerical simulation. Sun et al.[18] discussed the flow characteristics in a 90° bent pipe at different Reynolds numbers, and put forward the view that the higher the Reynolds number, the larger the pressure on the pipe wall. Stoesser et al.[19] compared the results of numerical simulation and experimental data for the flow and wall shear stress distributions in a meandering open channel. The emphasis of the aforementioned literature is on secondary flow in a curved channel or pipe, but research on the implantation of a stent in a curved vessel is sparse, therefore it is necessary to study the effects of secondary flow on the hemodynamics and drug distribution in curved vascular walls.

This paper applies the method of computational fluid dynamics (CFD) to investigate the effects on hemodynamics and drug concentration in a 3D 90° DES. At different Reynolds number the relationship between hemodynamics, drug concentration and in-stent restenosis was also studied. The results can provide a theoretical guidance for optimization of the design of DES.

## Methods

### Models

**Figure 1:**
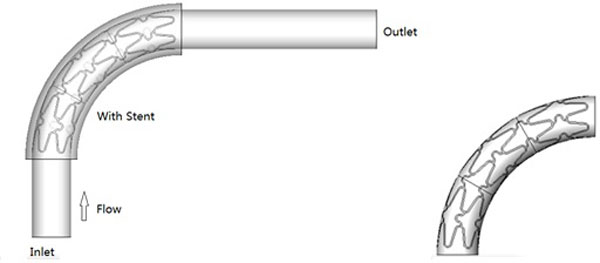
**Curved stent model**.

The configuration of the 90° stent, with six stent struts is shown in Figure 1). The radius of curvature is 10 mm. The diameter of the stent model is set as 3 mm, the length of stent is 15 mm, the thickness of the vascular wall is 0.5 mm, and the cross-section of the stent struts is square(0.10 mm × 0.10 mm). It was assumed that the stent makes full contact with the vascular wall. The Reynolds number are set as 200, 400, 600 and 800, with blood velocities of 0.22 m/s, 0.44 m/s, 0.66 m/s and 0.88 m/s, respectively. In order to ensure fully developed blood flow, a 30-mm length of straight tube was added at the inlet and outlet of the model.

GAMBIT 2.3 (ANSYS, Inc., USA) software was used to build the model. Unstructured meshes were applied to the model. Grids were refined at the stent and vascular surface to improve computational accuracy. The refinement on the meshes continued until the computational results reached grid-independence. A mesh number of about 3 million cells of the 90°curved stent was finally adopted in the following investigation.

### Boundary condition

Blood was assumed to be an isotropic and incompressible Newtonian fluid with constant density and viscosity and in steady flow. The vascular wall was assumed to be impermeable and rigid, and although not thoroughly correct, wall elasticity is of somewhat less importance in regard to the gross features of the flow. Previous studies strongly indicated that the effect of non-Newtonian fluid behaviour appears to be negligible[20,21]. Accordingly, the governing equations are as follows:

(1) $$\left\{\begin{array}{l} \frac{\partial u_{j}}{\partial x_{j}}=0\cdots \cdots \cdots \left(i,j=1,2,3\right)\\ \rho \frac{\partial u_{j}u_{i}}{\partial x_{j}}=-\frac{\partial P}{\partial x_{i}}+\mu \frac{\partial }{\partial x_{j}}\left(\frac{\partial u_{i}}{\partial x_{j}}+\frac{\partial u_{j}}{\partial x_{i}}\right) \end{array}\right)$$

where *ρ *and *μ *are the density and dynamic viscosity of blood, respectively, *ρ *= 1055 kg/m^3^, *μ *= 3.5 × 10^-3^kg/m·s; *P *is pressure, and *u_i _*is the velocity vector of blood.

Boundary conditions were set as follows. The parabolic profile with a centreline velocity was applied at the inlet; the range of inlet velocity is from 0.22 m/s to 0.88 m/s:

(2) $$\left\{\begin{array}{c} {\left(V_{z}\right|}_{inlet}=V_{m}\times \left(1-\frac{x^{2}+y^{2}}{r^{2}}\right)\\ {\left(V_{r}\right|}_{inlet}=0 \end{array}\right)=0.\text{22m}/\text{s},\;0.\text{44m}/\text{s},\;0.\text{66m}/\text{s and }0.\text{88m}/\text{s}$$

*V_z_*, *V_r _*are the velocities in the axial and radial directions of the blood flow, respectively. *r *= 0.0015m.

A zero pressure boundary condition was applied at the outlet.

(3) $${\left(P\right|}_{outlet}=0$$

Luminal drug transport was described by the steady-state convection-diffusion Eq. (4) and drug transport through the vascular wall was modelled by the steady state diffusion equation.

(4) $$\left\{\begin{array}{c} \frac{\partial }{\partial x_{i}}\left(u_{i}\phi_{f}-\tau_{kf}\frac{\partial \phi_{f}}{\partial x_{i}}\right)=0\\ \frac{\partial }{\partial x_{i}}\left(-\tau_{kt}\frac{\partial \phi_{t}}{\partial x_{i}}\right)=0 \end{array}\right)\begin{array}{ccc} & & \left(i=1,2,3\right) \end{array}$$

Φ_f_, Φ_t _are the drug concentrations in the blood and in the vascular wall, respectively. The diffusion coefficient of the drug through the blood (τ_kf_) is 10^-7^m^2^/s[21], and the diffusion coefficient of the drug through the tissue (τ_kt_) is 10^-12 ^m^2^/s[21].

(5) $${\left(\phi_{f}\right|}_{inlet}=0,{\left(\frac{\partial \phi_{f}}{\partial z}\right|}_{outlet}=0$$

(6) $${\left(\frac{\partial \phi_{t}}{\partial r}\right|}_{\begin{array}{cc} perivascular & wall \end{array}}=0$$

(7) $$\tau_{kf}{\left(\frac{\partial \phi_{f}}{\partial r}\right|}_{blood}=\tau_{kt}{\left(\frac{\partial \phi_{t}}{\partial r}\right|}_{tissue}\left(\text{Tissue}-\text{blood interface}\right)$$

(8) $$\frac{\partial \phi_{t}}{\partial z}=0\;\;\;\left(\text{Upstream and downstream boundaries of the tissue}\right)$$

(9) ϕ=1(1 indicates the stent surfaces assigned for drug coating)

The drug concentration was set to zero at the luminal inlet (Eq. 5), and an open boundary condition was applied at the distal boundary. The drug transport within the tissue was modelled as a simple diffusion process, with an impermeable boundary condition on the perivascular wall (shown in Eq. 6). The boundary condition of the continuity flux in drug transport is shown in Eq.7, which allows the entire drug in the blood at the tissue-blood interface to be transported into the arterial tissue. The drug release of the stents was simulated as a Dirichlet boundary condition (Eq. 9), with a drug concentration of unity at the strut surfaces.

The computations were conducted by using a commercial CFD package, FLUENT 6.3 (ANSYS Inc.), in which a finite volume method was used to discretize the governing equations. 3D single-precision format and a segregated solver were used. The SIMPLEC algorithm was applied to complete the velocity-pressure correction. The standard format was chosen for the pressure discretization and 2nd-order upwind for the momentum equations. The residual error convergence threshold was set as 0.0001.

**Figure 2:**
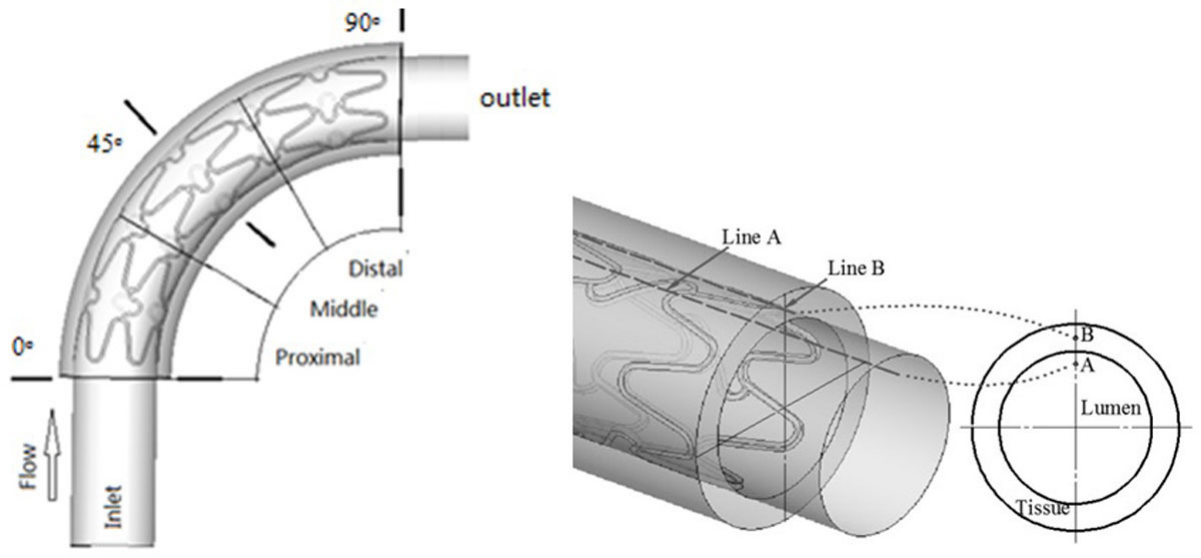
**Reference location (a)0°,45°,90° cross-sections (b) Reference Line A and Line B**.

To have a better observation on the drug concentration in the vascular wall for the curved stent, three planes were defined as the reference planes at 0°, 45°, 90° cross-sections from the × negative axis, clockwise(as shown in Figure 2(a)). Two lines in the middle segment were chosen for the investigation (as shown in Figure 2(b)). Line A is the reference line in the blood at a 1.25 mm radius, line B is another reference line at a 1.75 mm radius in the vascular wall.

## Results

### Secondary flow

In the case of Newtonian fluids, Dean Vortices appear as a pair of vortices in curved ducts or bends because of flow inertia. When fluid is directed around a curve under a pressure driven flow, the high velocity streams in the centre of the channel experience a greater centripetal force and so are deflected outward [22]. The Dean number (Dn) is a dimensionless group in fluid mechanics, which occurs in the study of flow in curved pipes and channels [23].

Dn = Re × (r/R)^1/2^

Re --- Reynolds number

r --- The diameter of the pipe

R --- Bend radius

When the Dean number is greater than 36, the fluid appears unstable phenomenon. The Reynolds numbers are 200, 400, 600, 800. In this paper, thus the Dean numbers are calculated as 77.5, 155, 232, and 310, respectively.

**Figure 3:**
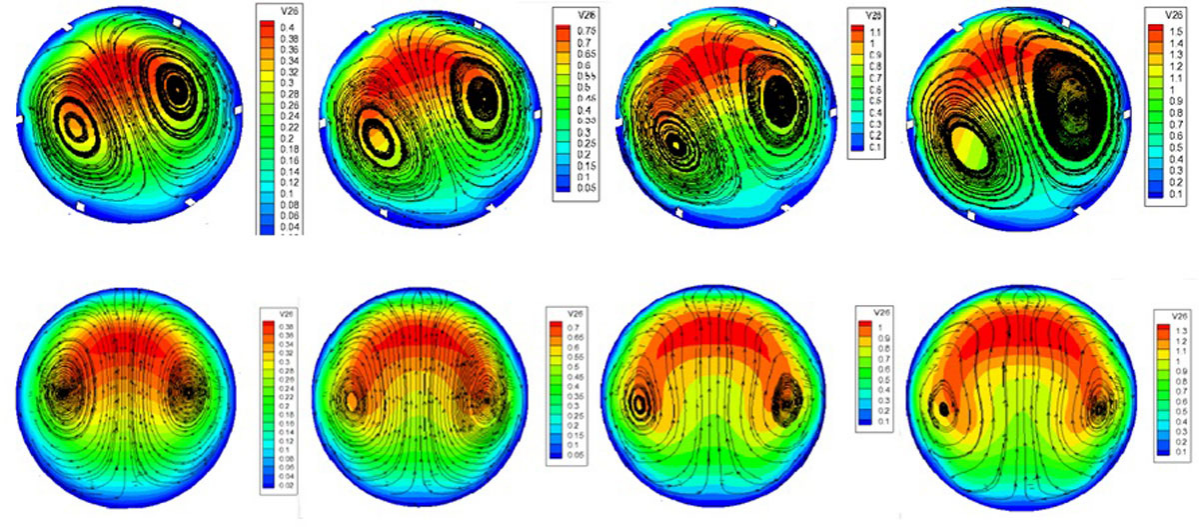
**The distributions of streamline on the cross section (a) 45°(b) 90°**.

The distributions of streamlines on the 45°and 90°cross-sections are shown in Figure 3, where the Dean vortex can be observed clearly. On the 45 °cross-section, the high velocity regions are mainly concentrated on the outer bend and the relatively low velocity regions are on the inner bend at small Reynolds numbers. With increasing Reynolds numbers, the high velocity regions reduce gradually in the middle of the pipeline, and close to both sides. Comparing the velocity on the 45 °and 90 ° cross-sections, the trend is consistent, but the velocity on the 90°cross-section is slightly smaller than that on the 45 ° cross section.

**Figure 4:**
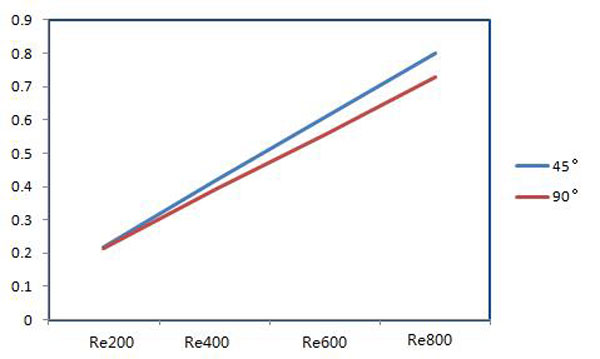
**Comparison of the peak values of velocity on the cross-sections**.

Comparison of the peak values of the velocity on the 45°and 90° cross-sections at different Reynolds numbers is shown as Figure 4. With increasing Reynolds numbers, the peak values have a linear growth. At a low Reynolds number, such as Reynolds number 200, the peak values are similar; the peak values on the 45°cross section are slightly higher than for on 90° at high Reynolds number.

### Velocity in cross-section

**Figure 5:**
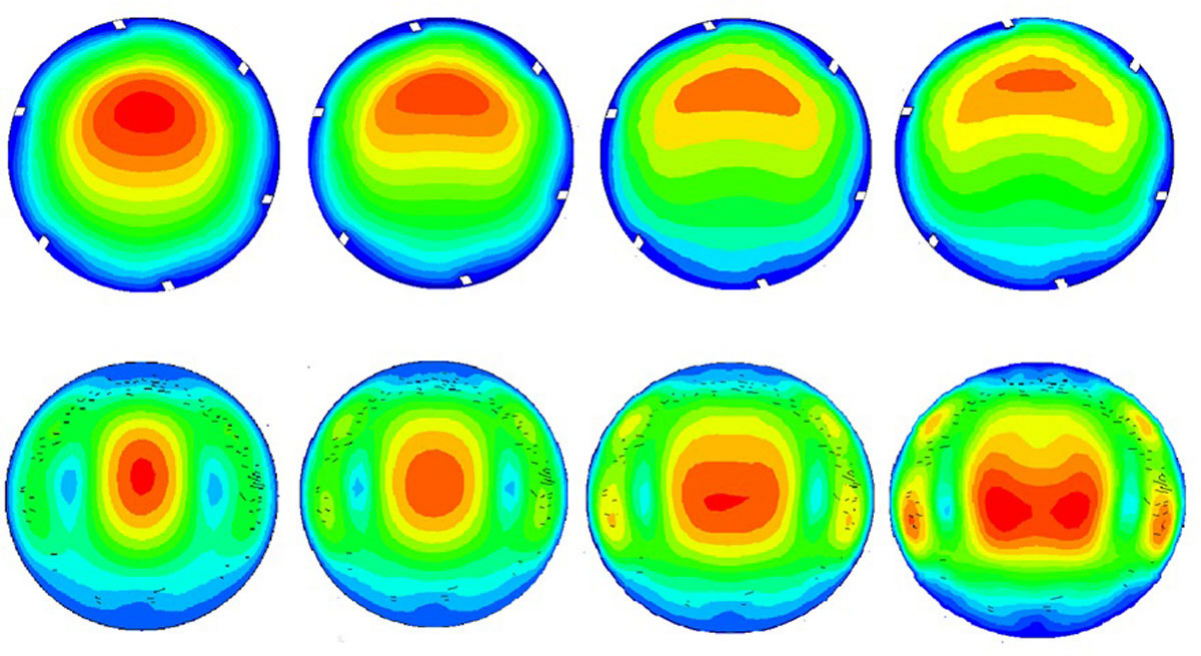
**The velocity on the cross sections (a) 45°(b) 90°**.

The distributions of the velocity on the 45°and 90° cross-sections for the model at different Reynolds numbers are shown in Figure 5. On the 45 ° cross-section, the high velocity region occurs in the centre of the axis and the relative area is large, and with increasing Reynolds number, the high velocity region moves to the outer bend side. On the 90° cross-section, when the Reynolds number is low, the high velocity region is small, and with the increase of the Reynolds number, the high velocity region slowly appears close to the two sides.

**Figure 6:**
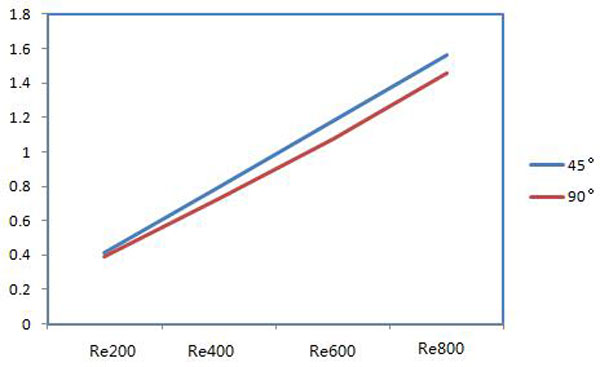
**The peak values of velocity on the cross sections**.

The peak values of velocity for these two-cross sections are shown in Figure 6. The peak values of velocity show the linear increases for both cross-sections and the values are bigger on 45°cross-section than that on 90°with increase of Reynolds number.

### WSS

**Figure 7:**
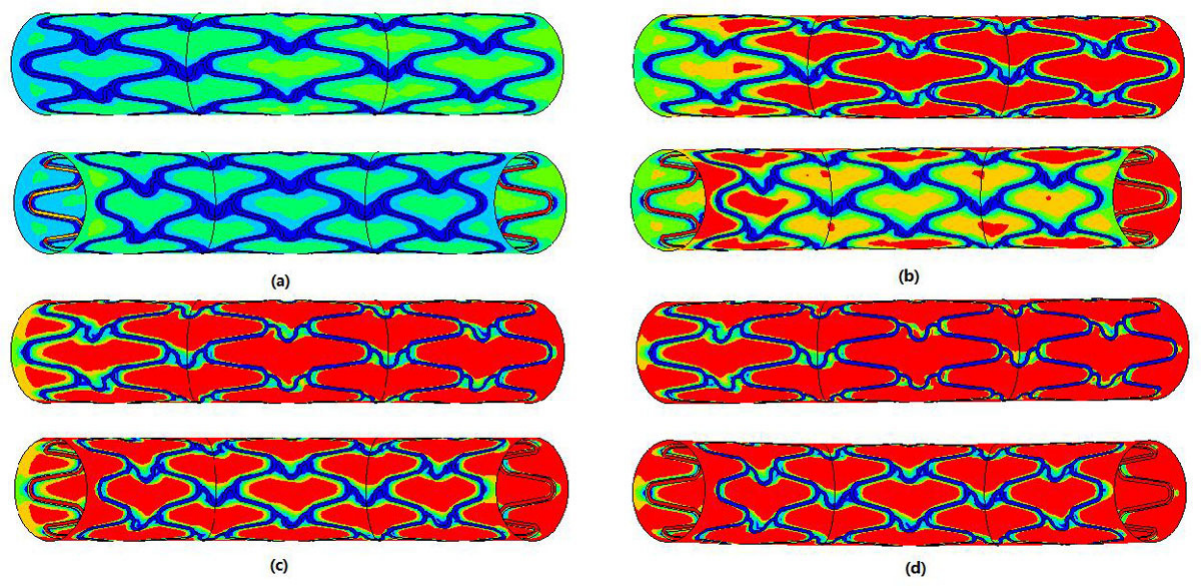
**The distributions of WSS for the model at different Reynold number**.

**Figure 8:**
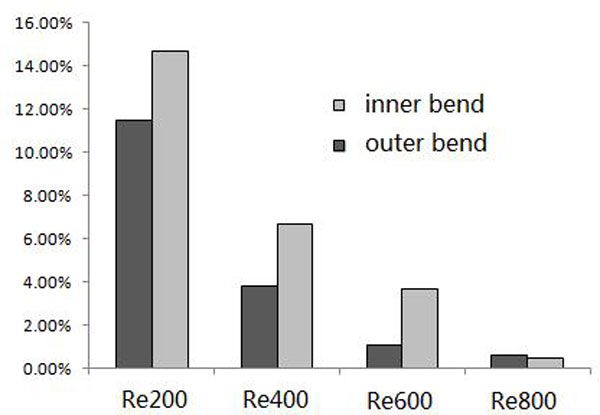
**The percentage of the low-WSS regions to the vascular wall surfaces for stent models with different Reynold number**.

The distributions of WSS for the model at different Reynolds numbers are shown in Figure 7. With increasing Reynolds number, low-WSS regions are fewer. A WSS of < 0.5 Pa is defined as a low WSS [20], shown as like dark blue region in Figure 7. The ratios of the low-WSS region to the whole model surface of the model at different Reynolds numbers are shown in Figure 8.

Obviously, the curved stent model at smaller Reynolds numbers has more low-WSS regions, which implies that it might be the most susceptible to in-stent restenosis. There are more low-WSS regions on the inner bend that on the outer bend. When the Reynolds number is larger than 600, the low-WSS ratio is less than 3%, which helps improve the in-stent restenosis.

### Drug concentration

**Figure 9:**
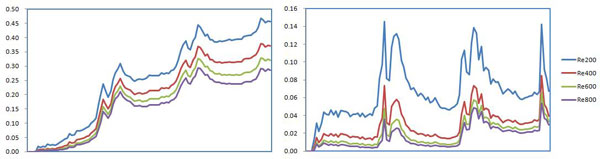
**The curves of drug concentration in blood (a) Inner bend (b) Outer bend**.

The drug concentration distributions on the inner/outer bends in Line A in the blood at different Reynolds numbers are shown in Figure 9. The peak value of drug concentration on the inner bend is 5 times higher than that on the outer bend and the average value on the inner bend is 10 times higher than that on the outer bend. The drug concentration on the inner bend reduces in proportion with the increase of blood flow velocity; the drug concentration at a Reynolds number of 200 on the outer bend is significantly higher than that at other Reynolds numbers. The phenomena of "Proximal low and distal high" are very obvious on the inner bend, with the value of drug concentration rising in each stent structs. The phenomena "Proximal low and distal high" is not obvious on the outer bend, where the drug concentration is flat between the stent struts.

**Figure 10:**
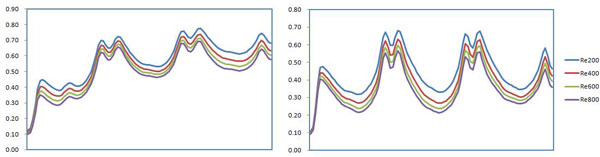
**The curves of drug concentration in the vascular walls (a)Inner bend (b)Outer bend**.

The drug concentration distributions on the inner/outer bends in Line B in the vascular walls at different Reynolds number are shown in Figure 10. The peak value of the drug concentration on the inner bend is 15% higher than that on the outer bend and the average value on the inner bend is 40% higher than that on the outer bend. The drug concentration on the inner bend doesn't obviously decreases compared with that on the outer bend in each stent structs. The drug concentration reduces proportionally with increasing Reynolds number, but the ratio of the drug concentration doesn't change much with Reynolds number.

**Figure 11:**
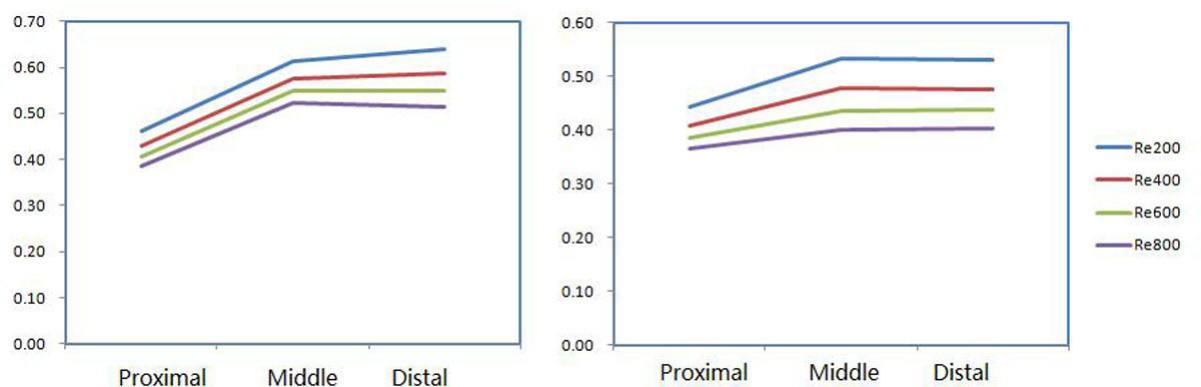
**The curves of drug concentration in proximal, middle and distal segments of the vascular walls (a)Inner bend (b)Outer bend**.

Comparisons of the mean values of the drug concentration in the proximal, middle and distal segments are shown in Figure 11. The drug concentration increases gradually with blood flow direction, and the drug concentration in the distal is higher than that in the proximal and middle in a straight pipe [21]. For the curved pipe, secondary flow is a problem that can't be avoided. Due to the effects of secondary flow in the curved stents, the velocity in the circumferential direction helps gather and accumulate the drug in the blood in the middle and distal segments of the stent. The drug concentration in middle segment at low Reynolds number is slightly higher than that in distal on the inner bend. On the contrary, the drug concentration in the distal is slightly higher than that in the middle at high Reynolds number; the drug concentrations in the distal and in the middle are almost uniform on the outer bend. It can be seen that the drug concentration is more obvious on the outer bend in the middle and distal of the stent with increase of Reynolds number.

## Discussion

Atherosclerosis in carotid arteries and coronary arteries, abdominal aorta, renal artery and aortic arch, after DES implanted in blood vessels, causes the velocity of the blood flow to be different in different parts of the human body. There is a lack of quantitative researches on the effects of the WSS and drug concentration in the blood and vascular wall at different Reynolds number.

Some diseases may make the blood flow velocity faster or slower, dynamic changes in the blood viscosity would lead to changes in the blood velocity. The elderly are susceptible to hyper viscosity, and there are many factors that affect the blood viscosity: blood cell factors, such as blood cells number, size, shape, RBC deformability, platelet function, etc; plasma reasons, such as plasma proteins (especially fibrinogen, immune globulin), blood glucose, blood lipid, fibrin lytic activity, etc; vascular factors, such as length, diameter and lining smoothness, etc; other factors, such as emotions, life style, smoking, drinking, etc. Common features of these diseases, such as atherosclerosis, moyamoya disease, vasculitis, partial recanalization, inflammation, and tumor thrombus, are caused by vascular stenosis. Radioactive damage caused by artery stenosis, dissecting aneurysm, and vasospasm is one of the reasons causing artery stenosis. For healthy human beings there are similar situations, the velocity of the blood flow changes according to the different statuses of the body: when undertaking sports, the velocity of the blood flow will be faster; when at rest and the velocity of the blood flow will be relatively slower.

We can see clearly the effects of Reynolds number on the WSS and drug concentration. When the Reynolds number increases, the low-WSS region gradually decreases, for a Reynolds number of 600, the percentage of the low-WSS region is less than 3%, and the Reynolds number of 800, the percentage decreases to 1%, with the effect of low-WSS almost negligible. Therefore, as the Reynolds number increases, the low-WSS region decreases.

Increasing blood flow velocity leads to a decrease of the drug concentration in the vascular walls, for example, increasing Reynolds number to 400 from 200, the drug concentration drops 8% approximately, but increase to 800 from 200 that is four times of original value 200, causes the drug concentration to decrease by 30% approximately. The effects of decreasing drug concentrations on the outer bend are greater than that on the inner bend.

## Conclusions

In this study, the effects of different Reynolds number on the hemodynamics, drug concentration and secondary flow were analyzed with 3D DES models. The results showed that:

(1) The intensity of secondary flow on the 45° cross-section was stronger than that on the 90° cross-section, meaning that the enhancement of flow in the circumferential direction leads to a decrease of the low-WSS region;

(2) With increasing Reynolds number, the region of low-WSS decreased. When the Reynolds number is larger than 600, the region of low-WSS is smaller than 3%.

(3) The effects of Reynolds number on drug concentration in the vascular wall decreased proportionally and as blood velocity increased 4 times, the drug concentration in the vascular wall decreased by 30%.

(4) As the blood velocity increased, the drug concentration in the DES decreased, especially on the outer bend.

When the DES is implanted in the blood vessel, it is beneficial for the patient to decrease vigorous activities and keep calm at the beginning of stent implantation, because a substantial amount of drug is released in the first two months of stent implantation, and calmness is conducive to drug release and absorption. Later, appropriate exercise can achieve higher blood velocity which is helping in decreasing regions of low-WSS.

## Competing interests

Other than the grants listed in the acknowledgement section, the authors declare that they have no competing interests.

## Authors' contributions

YC and YX conceived of the study, developed and implemented the modelling, calculation and analysis, and wrote most of the manuscript. WJ conceived the study, contributed to the whole frame, and wrote parts of the manuscript. FY and MG supplied the data and rigorously revised the manuscript. QW and YF thoroughly reviewed the manuscript.

Yu Chen and Yan Xiong contributed equally to this work and should be considered as co-first authors.

## Declarations

Publication of this article was paid with funding from Grants-in-Aid from the National Natural Science Research Foundation of China (No. 61190123, 51205262, 10872138) and Applied Basic Research Programs of science & technology department of Sichuan Province (No. 2014JY0148).

This article has been published as part of *BioMedical Engineering OnLine *Volume 14 Supplement 1, 2015: Cardiovascular Disease and Vulnerable Plaque Biomechanics. The full contents of the supplement are available online at http://www.biomedical-engineering-online.com/supplements/14/S1
